# The Annealing of Acetylated Potato Starch with Various Substitution Degrees

**DOI:** 10.3390/molecules26072096

**Published:** 2021-04-06

**Authors:** Tomasz Zięba, Aleksandra Wilczak, Justyna Kobryń, Witold Musiał, Małgorzata Kapelko-Żeberska, Artur Gryszkin, Marta Meisel

**Affiliations:** 1 Department of Food Storage and Technology, Faculty of Biotechnology and Food Science, Wroclaw University of Environmental and Life Sciences, Chełmońskiego 37, 51-630 Wrocław, Poland; tomasz.zieba@upwr.edu.pl (T.Z.); artur.gryszkin@upwr.edu.pl (A.G.); marta.meisel@upwr.edu.pl (M.M.); 2 Department of Physico-Chemistry of Microorganisms, University of Wroclaw, Przybyszewskiego 63-77, 51-148 Wrocław, Poland; aleksandra.wilczak@uwr.edu.pl; 3 Department and Chair of Physical Chemistry and Biophysics, Wrocław Medical University, Borowska 211A, 50-556 Wrocław, Poland; justyna.kobryn@umed.wroc.pl (J.K.); witold.musial@umed.wroc.pl (W.M.)

**Keywords:** annealing, acetylation, starch particle size distribution

## Abstract

This study aimed to determine the effect of “annealing” acetylated potato starch with a homogenous granule size and various degrees of substitution on the thermal pasting characteristics (DSC), resistance to amylases, rheology of the prepared pastes, swelling power and dynamics of drug release. A fraction of large granules was separated from native starch with the sedimentation method and acetylated with various doses of acetic anhydride (6.5, 13.0 or 26.0 26 cm^3^/100 g starch). The starch acetates were then annealed at slightly lower temperatures than their pasting temperatures. The annealing process caused an almost twofold increase in the resistance to amylolysis and a threefold increase in the swelling power of the modified starch preparations. The heat of phase transition decreased almost two times and the range of starch pasting temperatures over two times, but the pasting temperature itself increased by ca. 10 °C. The 40 g/100 g addition of the modified starch preparation decreased the rate of drug release from a hydrogel by ca. one-fourth compared to the control sample.

## 1. Introduction

The process of native starch heating in a water suspension having a temperature that fits within the range of glass transition temperatures and the initial pasting temperatures is called annealing. The goal of this thermal modification is to bring starch temperature to such a value that would excite the motility of starch molecules but prevent starch transformation into a paste. It makes the crystalline structure of starch homogeneous, thereby protecting the structure of granules [1,2,3]. Annealing causes an increase in pasting temperatures and, at the same, makes the temperature range narrower. In addition, it has a little effect on process enthalpy. If starch retains its grainy structure after modification, its swelling power decreases with an increase in pasting temperatures [4]. In turn, the viscosity of pastes obtained from annealed starch depends on starch origin. For example, the annealing process was reported to increase the viscosity of pastes made of wheat and potato starches, and to decrease the viscosity of those obtained from lentil and oat starches [5]. Starch modified using this type of heat treatment has some special applications in the food industry. Due to its thermal stability and reduced tendency for retrogradation, it is used in food preserves and frozen foods [6]. In turn, Hormdok R. and Noomhorm A. [7] showed the hydrothermally-modified starch exhibited properties desirable in pasta making. The annealing can also contribute to an increased content of resistant starch, especially when coupled with other modifications [4]. The double-modified starch can also have some non-food uses. The application of various modified biopolymers may lead to the development of new hydrogel formulations to be used in the field of drug delivery [8].

The annealed native starch of various botanical origin has been broadly investigated and described [9,10]. In turn, few works are available regarding changes evoked by annealing in the earlier chemically-modified starch [11,12,13]. Potato starch proves to be an excellent raw material for chemical modifications because, unlike cereal starches, its granules do not contain other substances, such as protein and fat [14]. In the case of potato starch, small changes in the chemical structure of the polymer cause significant modifications in its properties [15]. Despite a high chemical purity, potato starch is not a homogenous material. Its pasting temperature depends on, i.a., the size of its granules [14], which ranges from a few to over 100 µm. In the case of its annealing, this property triggers differences in the transformations of granules having various sizes. To minimize the effect of this factor, it seems reasonable to use the fraction of granules obtained by sorting native potato starch.

The aim of this study was to determine how the annealing of acetylated starch with homogeneous granule size can affect its thermal pasting characteristics (DSC), resistance to amylases, rheology of the prepared pastes, swelling power and drug release dynamics.

## 2. Results

**Figure 1:**
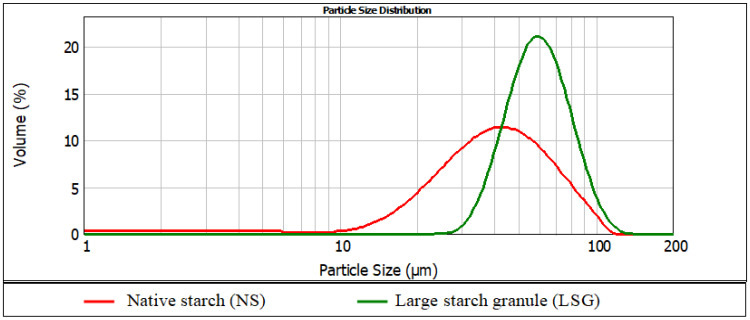
Particle size distribution of native starch (NS) and starch with homogenous fraction size determined using a laser particle size diffraction analyzer (LSD).

Potato starch granules differ significantly in size; their diameters can range from a few to over 100 µm. Granules of various sizes undergo pasting at different temperatures and for this reason the raw material used in our study to produce annealed acetylated starch was firstly homogenized in terms of granule size. Particle size distribution of native starch and of native starch with homogenized fraction size determined using a laser particle size diffraction analyzer (LSD) is presented in Figure 1.

The mean granule diameter of the non-homogenized starch, expressed as the volumetric diameter D[4,3], reached 42.3 μm, and that measured after separating small granules reached 61.7 μm. In the present study, the acetylation of large starch granules with various doses of acetic acid anhydride resulted in the production of starch acetates which differed significantly in the degree of substitution and, consequently, in the initial pasting temperatures (Table 1).

The dependency between starch acetylation and the resulting decrease in pasting temperatures, including the greater decrease along with an increasing degree of starch substitution with acetic acid residues, has been extensively documented in literature [16,17,18,19]. For this reason, the modified starch preparations were subjected to the annealing treatment at various temperature, however in each case the annealing temperature was only slightly lower than their initial pasting temperature.

**Figure 2:**
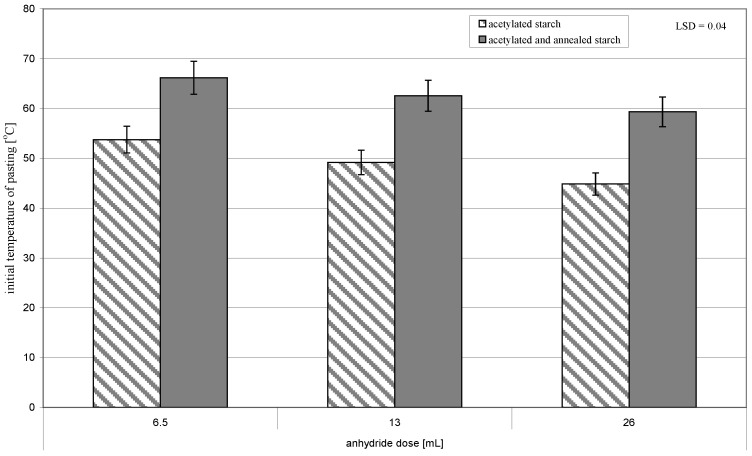
Initial temperature of pasting of preparations of acetylated starch and of acetylated and annealed starch.

**Figure 3:**
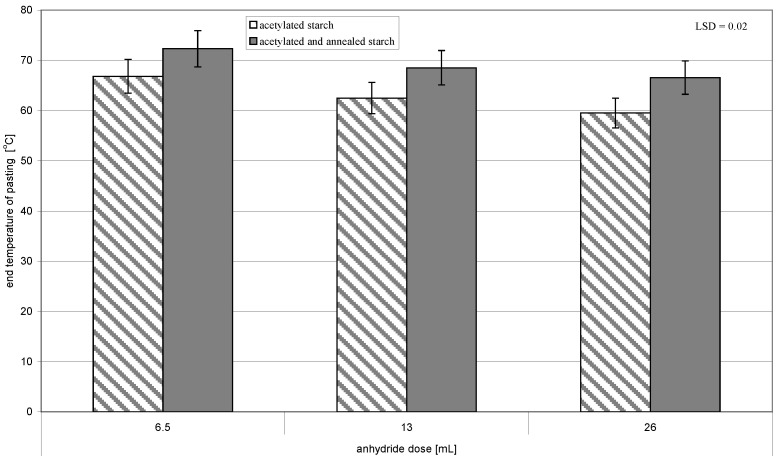
End temperature of pasting of preparations of acetylated starch and of acetylated and annealed starch.

**Figure 4:**
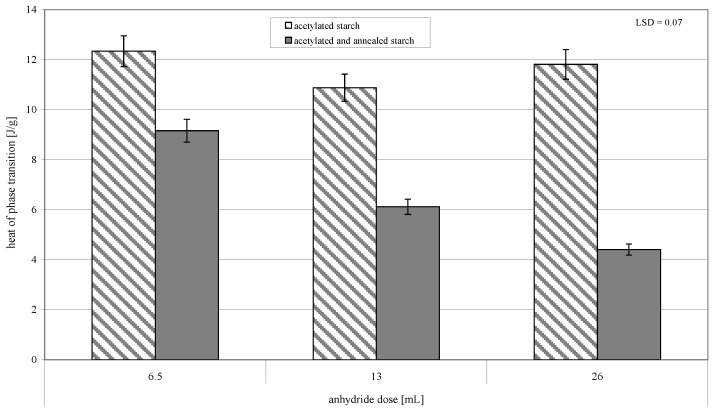
Heat of the phase transition of preparations of acetylated starch and of acetylated and annealed starch.

The annealing of acetylated starch led to a significant increase in the initial (Figure 2) and final temperature of pasting (Figure 3). It also caused the narrower range of temperatures and the lower heat of this process (Figure 4). The observed changes were promoted by an increasing acetylation degree of the annealed starch preparations. According to Adebowale et al. [6], an increase in pasting temperatures reflected the melting of crystallites, which are formed during interactions of amylose-amylose and amylose-amylopectin chains as a result of heating induced by native starch structure reorganization, and which caused its crystallinity to increase. It needs to be remembered that in the present study we deal not with natural starch but with acetylated starch, in which bonds between chains were weakened due to the replacement of hydroxyl groups with acetyl groups imparting the amphiphilic character to starch [20]. Acetylation of starch with an acetic anhydride proceeded mainly in the outer layers of starch granules [21]. Therefore, probably, its interior underwent similar processes during annealing as the native starch did. Hence, the changes in the thermal pasting characteristics determined with DSC were due to the effects of acetylation and annealing, and their magnitude depended on conditions of both these processes.

**Figure 5:**
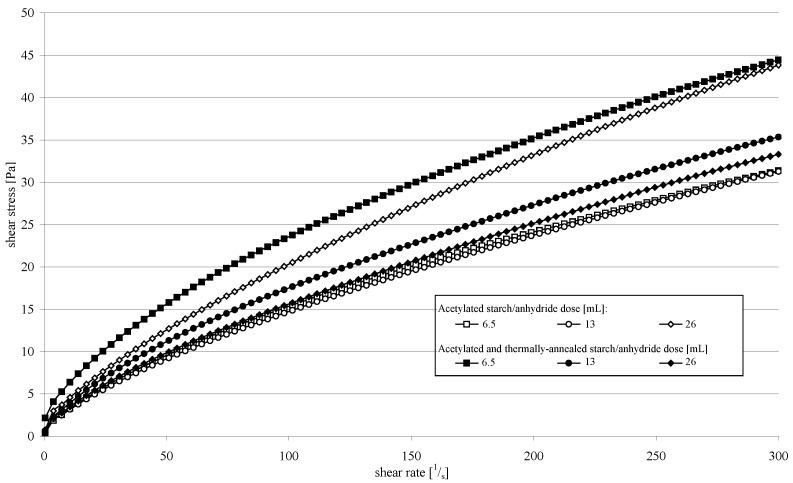
Flow curves of starch pastes prepared from acetylated starch and from acetylated and thermally-annealed starch.

Figure 5 presents flow curves of starch pastes made from acetylated starch and from acetylated and thermally-annealed starch. Among the preparations which were not subjected to the annealing process in the entire course of the flow curve, the highest viscosity was noted for the paste prepared from acetylated starch with the highest degree of substitution (DS). Starch esters with a lower DS formed less viscous pastes. Analogous tendencies were observed in the values of rheological coefficients (Table 1). Similar dependencies were previously reported in acetylated potato starch by many authors [19,22,23]. The annealing of acetylated starch contributed to opposite dependencies. In the whole course of the flow curve, the highest viscosity was noted for the paste prepared from starch acetylated with the lowest dose of the acetic acid anhydride, whereas the lowest viscosity was for the paste made of starch acetylated with the highest anhydride dose. The reversal of tendencies also applied to the values of rheological coefficients. In the case of native starch, annealing increases paste viscosity [5]. At a low degree of esterification, the viscosity of the starch paste increased due to hydrothermal hardening the same as in the case of natural starch. In the case of starch esterified with the highest dose of acetic anhydride, it can be assumed that the greatest hydrolytic changes in the starch structure occurred. The consequence of these changes was probably the flowing of more starch substance into the solution during annealing compared to the other preparations. Large changes in the structure of this starch could reduce the viscosity of the prepared starch paste.

**Figure 6:**
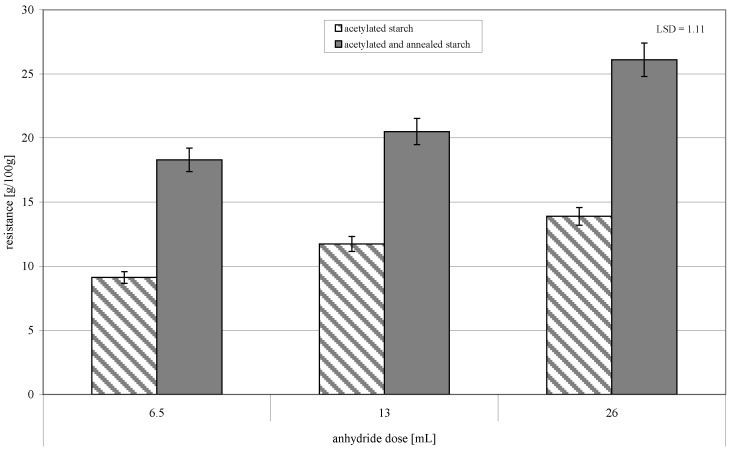
Resistance to amylolysis of preparations of acetylated starch and of acetylated and annealed starch.

The resistance of starch acetylated with various doses of acetic acid anhydride to amylolysis increased from a few to dozen per cents along with an increasing DS of starch acetates (Figure 6). The annealing caused ca. a twofold increase in starch resistance. Starch acetylation contributes to its partial resistance to amylolysis [24] because the attached ester groups hamper enzyme’s access to the bond being hydrolyzed [25]. Hydrothermal modifications, like e.g., heat-moisture treatment (HMT) or annealing, also increase the content of resistant starch in a starch granule [26,27,28,29]. The earlier described interactions of amylose and amylopectin impair the enzymatic hydrolysis [4,30,31]. Starch resistance can also be increased by the elution of amylolysis-susceptible low-molecular-weight chains from the amorphous region of starch granules, which leads to an increased content of the resistant fractions in a starch granule.

**Figure 7:**
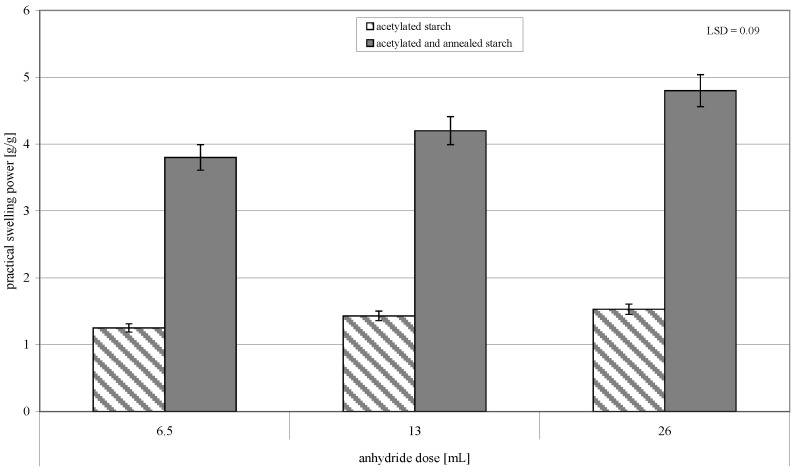
Practical swelling power of acetylated and of acetylated and annealed starch preparations.

The annealing of native starch induces changes in its swelling powers. Trends and magnitude of these changes depend on the botanical origin of starch and process parameters [32,33,34,35]. The heating of large granules of acetylated starch at temperatures below its pasting temperature led to ca. a threefold increase in the practical swelling power of the modified preparations (Figure 7). Values of this parameter increased along with an increasing acetylation degree of the double-modified starch preparations. This increase could have been due to a higher number of acetyl groups incorporated into the starch chain, which decreased the strength of binding between starch molecules, thereby increasing their swelling power [35,36]. An additional factor that increased the swelling power of the starch preparations could have been the homogenized granule size of starch. The simultaneous swelling during heating in water and the release of low-molecular-weight chains from all starch granules increase granules’ porosity [37] and, consequently, the swelling power of the modified preparation.

**Figure 8:**
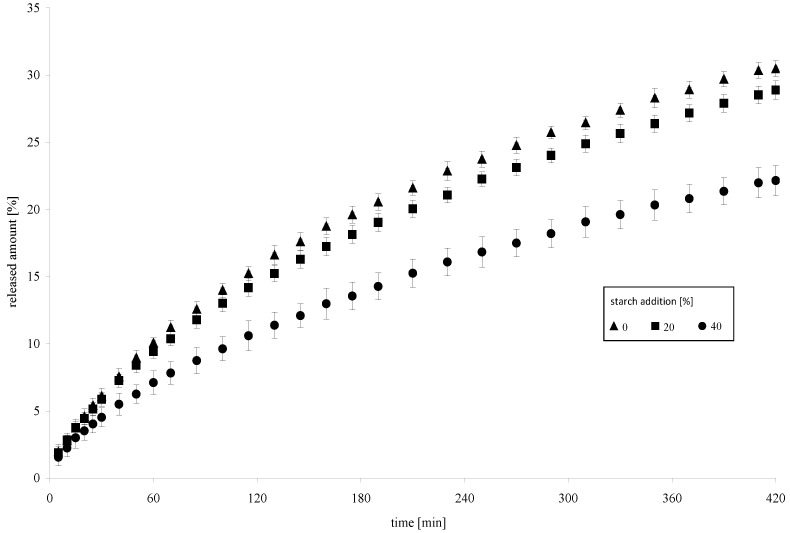
Dynamics of a model drug release from a hydrogel containing modified starch.

The modified properties of annealed acetylated starch prompted us to perform a pilot experiment to identify possibilities of the practical application of starch preparations modified in this way. Many authors work on bioderivative polymers used in the controlled drug delivery [35]. Figure 8 presents the dynamics of a model drug release from a hydrogel containing modified starch. The 20 g/100 g addition of the modified starch preparation caused a relatively small change in the drug release dynamics compared to the gel produced without starch. At the first stage of the process, differences in the amount of extracted drug were negligible, whereas after 420 min–the difference reached 5.2 g/100 g compared to the control sample. Significantly greater differences were observed for the hydrogel produced with 40 g/100 g addition of the modified starch. As soon as after 30 min, the difference in drug extraction rate was 26.2 g/100 g compared to the control hydrogel (H0) and maintained at a similar level until the end of the experiment, to finally reach 27.5 g/100 g after 420 min. Results of this pilot experiment allowed us to conclude that the use of modified starch can enable developing new formulations for the controlled local drug delivery. This will, however, require further specialized research.

## 3. Materials and Methods

### 3.1. Materials

Experimental materials included: Superior Standard potato starch manufactured by PEPEES Łomża S.A. Acetic anhydride was purchased at POCH SA Gliwice company.

### 3.2. Production of Modified Preparations

A fraction of large granules was separated from native potato starch with the sedimentation method. A suspension was made from 5.3 g of starch per 100 g of the solutions in a 15-L vessel, with the height of the liquid column reaching 19.5 cm. After thorough stirring, the suspension was left for 3 min. Then, the precipitate was decanted and the missing volume was completed with distilled water. Another sedimentation and decantation cycle was repeated three times more. The precipitate of large granules was air-dried at a temperature of 30 °C for 48 h. Native starch and starch left after the separation of the large granule fraction were analyzed using a laser particle size analyzer (Malvern Instruments LTD, UK) with a Hydro 2000 attachment.

The large granule fraction was acetylated with the basic dose of acetic acid anhydride used for potato starch modification under industrial conditions (13 mL/100 g starch) [38] and with its 0.5-fold and 2-fold doses (6.5 or 26 mL/100 g starch). The acetylated starch was rinsed with distilled water until reagent residues were removed, dried at 30 °C for 24 h, ground, and sieved through a screen with mesh size of 400 μm.

The pasting characteristics (DSC) of acetylated starch was used to establish the initial pasting temperature, which was further used to selected annealing temperatures. A suspension from 10 g of starch/100 g of the solutions was prepared in a 2-L vessel from starch acetylated with three doses of the acetic acid anhydride (6.5, 13 or 26 mL/100 g starch). Under continuous stirring, the suspension was heated (water bath with stirrer) to the temperatures of 52 °C, 48 °C or 43 °C (for respective anhydride doses) with stirring speed 240 rpm for 8 h and left for 48 h. Afterward, it was rinsed three times with 5-L portions of distilled water. Starch precipitate was separated each time from the suspension using a Stratos flow centrifuge (Heraeus, Sepatech, Germany), then air-dried at 30 °C for 24 h, ground in a mortar, and sieved through a screen with mesh size of 400 μm.

### 3.3. Determination of the Degree of Acetylation of Starch Preparations

A suspension of 10 g of starch was prepared in 65 cm^3^ of distilled water and stirred. Then, phenolphthalein was added and a 0.1 M NaOH solution was instilled to obtain a light-pink color of the mixture that maintained for 1 min. Next, 25 cm^3^ of a 0.5 N NaOH solution were added and the reaction mixture was stirred on a shaker for 35 min. Afterward, it was titrated with a 0.5 M HCl solution with a known titer [8].

### 3.4. Determination of the Characteristics of Phase Transitions of Starch Preparations with Differential Scanning Calorimetry (DSC)

This determination was conducted using a DSC 822E differential scanning calorimeter (Mettler Toledo, Giessen, Germany). Before the measurement, the calorimeter was calibrated using and indium sample and a zinc sample. Pre-tests were performed in a temperature range of 0–100 °C. Aluminum crucibles (100 μL) with lids were used for analyses. A 10-g starch sample weighed exact to ± 0.02 mg was placed on crucible’s bottom, and then distilled water was added in the ratio of 3:1 respective to sample weight. The measuring crucible was covered with a lid, conditioned at 25 °C for 30 min, transferred to an oven chamber having a temperature of 25 °C, and heated to a temperature of 100 °C at the heating rate of 4 °C/min. The initial and final temperatures of pasting, and the heat of this transition were determined from thermograms [39,40].

### 3.5. Determination of the Flow Curves of Pastes Made of Starch Preparations Using a Haake Oscillating-Rotating Viscosimeter 

Analyses were carried out using an RS 6000 oscillating-rotating viscosimeter Haake (Karlsruhe, Germany) for 5 g/100 g starch suspensions that were heated at 96 °C for 30 min under continuous stirring [41].

Flow curves were plotted for the pastes at a measurement temperature of 50 °C and shear rate of 1–300 s^−1^. A hot paste was placed in a system of coaxial cylinders (Z38AL type) of an RS 6000 rheometer, then cooled, and relaxed at the measurement temperature for 15 min. The flow curves plotted were described using the following equations:

Oswald de Waele:

τ = K × γ^n^ (1)

Casson:

τ = τ_0C_^0.5^ + (η_C_ × γ)^0.5^ (2)

where: τ–shear stress (Pa), K–consistency coefficient (Pa∙sn), γ–shear rate (s^−1^), n–flow index, τ_0C_–yield point (Pa), η_c_–Casson’s plastic viscosity (Pa∙s).

### 3.6. Determination of the Resistance of Starch Preparations to Amyloglucosidase Action

A 0.72 g/100 g suspension was prepared from 38 g of starch that was heated to the boiling point and then cooled. The volume of evaporated water was completed, and then 34 mL of an acetate buffer and 4 mL of an amyloglucosidase solution (Genecor) were added. Thus prepared sample was hydrolyzed in a water bath at a temperature of 37 °C. Enzyme concentration was adjusted to ensure the complete saccharification of native starch after 120 min of the process. The amount of released glucose was controlled every hour. To this end, 1-mL samples were collected, centrifuged and transferred in 10-µL portions to 2-mL cuvettes containing 1 mL of the reagent for glucose concentration determination (BIOSYSTEM), stirred and incubated at 20 °C for 15 min. Then, absorbance was measured at a wavelength of λ = 500 nm using a CECIL CE 2010 colorimeter (Cecil Instruments, Cambridge, United Kingdom). Glucose content was read out from the standard curve plotted as above with glucose solutions. Three results that did not differ from each other were acknowledged as the moment of complete saccharification of starch [41].

### 3.7. Determination of Practical Swelling Power of Starch Preparations in Water Having a Temperature of 20 °C

Starch (300 mg) was weighed into a 4-mL test tube, then 2.5 mL of distilled water were added and the sample was conditioned at a temperature of 20 °C for 10 min. Next, the sample was centrifuged using an MPW-55 laboratory centrifuge (MPW Instruments, Warsaw, Poland) at 5000 rpm for 10 min. Afterward, supernatant was collected and the precipitate left in test tubes was dried with a filter paper and weighed. The swelling power was expressed in grams of water absorbed by one gram of starch dry matter.

### 3.8. Determination of the Dynamics of Model Drug Release from a Hydrogel with the Addition of a Modified Starch Preparation

These analyses were conducted with starch acetylated using the basic dose of acetic acid anhydride (13 mL/100 g starch) and annealed. Selected modified starch batches (20 g/100 g or 40 g/100 g) were used to produce hydrogels with 1.5 g/100 g of polyacrylate crosspolymer 11 (PC-11, Aristoflex Velvet, Clariant, Muttenz, Switzerland) as the vehicle, and with 12 g/100 g of a dense extract of chestnut seeds (extractum spissum hippocastani, EH, WZZ Herbapol SA, Wrocław, Poland) as the model drug. The formulation contained water was the completive component. The hydrogels shortened, respectively, as H20 and H40 were used to evaluate the drug release from formulations containing modified starch. Hydrogel of the same composition, containing water instead of starch, was established as the control–H0. The release study for the selected modified starch batches was performed acc. to the pharmacopoeial method with extraction cells, and purified water applied as a dissolution medium (Drug Dissolution Tester Erweka GmbH DT 700, Heusenstamm, Germany) [Ph. Eur. 9.0, 20903 (01/2016)]. The released drug amount was assessed via spectrophotometry (Spectrophotometer UV/VIS Jasco V-530, Tokyo, Japan) according to the available bibliography [42,43].

### 3.9. Statistical Analysis

Results were statistically analyzed using Statistica 13.3 PL software package. Based on statistical computations (from at least three parallel replications), values of the least significant differences and standard deviation were calculated and equations of flow curves were determined. For statistical evaluation, the results were subjected to one-way analysis of variance at a significance level of 0.05. Values of the least significant difference (LSD) between the means were computed using the Duncan’s test at a significance level of 0.05 (StatSoft, Inc., Tulsa, OK, USA, 2011).

## 4. Conclusions

The annealing of large granules of acetylated starch caused an increase in pasting temperatures, starch resistance to amylolysis and swelling power, but also a decrease in the heat of modified starch pasting. The magnitude of the observed changes depended on starch acetylation degree. The viscosity of pastes made from double-modified starch was determined mainly by the degree of starch acetylation. The annealing of starch with a lower DS caused an increase, whereas that of starch with the highest DS caused a decrease in the viscosity of the pastes. The 40% addition of modified starch decreased the rate of drug release from the hydrogel by 25% compared to the control sample. This promising discovery deserves in-depth investigations to identify its potential applicability in the pharmaceutical industry.

## Figures and Tables

**Table 1:** Degree of substitution and initial temperature of pasting of large granules of starch acetylated with various doses of an acetic acid anhydride.

Acetic Acid Anhydride Dose [mL]	Acetylation Degree []	Initial Temperature of Pasting [°C]
6.5	0.05 ± 0.001	53.8 ± 0.01
13	0.11 ± 0.001	49.2 ± 0.02
26	0.18 ± 0.001	44.9 ± 0.01
LSD	0.01	0.03

Mean values from three replications ± standard deviations, LSD-least significant differences.

## Data Availability

Data is contained within the article.

## Sample Availability

Samples of the compounds are not available from the authors.

## References

[1] Din Z.U., Xiong H., Fei P. (2015). Physical and Chemical Modification of Starches-A Review. Crit. Rev. Food Sci. Nutr..

[2] Rocha T.S., Felizardo S.G., Jane J., Franco C.M.L. (2012). Effect of annealing on the semicrystalline structure of normal and waxy corn starches. Food Hydrocoll..

[3] Piecyk M., Konarzewska M., Sitkiewicz I. (2009). Wpływ modyfikacji hydrotermicznej typu annealing na wybrane właściwości skrobi grochu (Pisum sativum). Żywność. Nauka. Technologia. Jakość.

[4] Zavareze E.D.R., Dias A.R.G. (2011). Impact of heat-moisture treatment and annealing in starches: A review. Carbohydr. Polym..

[5] Tester R.F., Debon S.J.J. (2000). Annealing of starch—A review. Int. J. Biol. Macromol..

[6] Adebowale K.O., Afolabi T.A., Olu-Owolabi B.I. (2005). Hydrothermal treatments of Finger millet (Eleusine coracana) starch. Food Hydrocoll..

[7] Hormdok R., Noomhorm A. (2007). Hydrothermal treatments of rice starch for improvement of rice noodle quality. LWT-Food Sci. Technol..

[8] Van Vlierberghe S., Dupruel P., Schact E. (2011). Biopolymer-Based Hydrogels As Scaffolds for Tissue Engineering Applications: A Review. Biomacromolecules.

[9] Wang S., Jin F., Yu J. (2012). Pea Starch Annealing: New Insights. Food Bioproc. Tech..

[10] Wang S., Wang J., Wang S., Wang S. (2017). Annealing improves paste viscosity and stability of starch. Food Hydrocoll..

[11] Nakazawa Y., Wang Y.J. (2004). Effect of annealing on starch–palmitic acid interaction. Carbohydr. Polym..

[12] Masina M., Choonara Y.E., Kumar P., du Toit L.C., Govender M., Indermun S., Pillay V. (2017). A review of the chemical modification techniques of starch. Carbohydr. Polym..

[13] Majzoobi M., Sabery B., Farahnaky A., Karrila T.T. (2012). Physicochemical properties of cross-linked-annealed wheat starch. Iran Polym. J..

[14] Dupuis J.H., Liu Q. (2019). Potato Starch: A Review of Physicochemical, Functional and Nutritional Properties. Am. J. Potato Res..

[15] Zięba T., Solińska D., Kapelko-Żeberska M., Gryszkin A., Ačkar Đ., Lončarić A., Babić J., Jozinović A. (2020). Properties of roasted starch with apple distillery wastewater. Polymers.

[16] Bello-Pérez L.A., Agama-Acevedo E., Zamudio-Flores P.B., Mendez-Montealvo G., Rodriguez-Ambriz S.L. (2010). Effect of low and high acetylation degree in the morphological, physicochemical and structural characteristics of barley starch. LWT-Food Sci. Technol..

[17] Diop C.I.K., Li H.L., Xie B.J., Shi J. (2011). Effects of acetic acid/acetic anhydride ratios on the properties of corn starch acetates. Food Chem..

[18] Kapelko M., Zięba T., Michalski A. (2012). Effect of the production method on the properties of RS3/RS4 type resistant starch. Part 2. Effect of a degree of substitution on the selected properties of acetylated retrograded starch. Food Chem..

[19] Mbougueng P.D., Tenin D., Scher J., Tchiégang C. (2012). Influence of acetylation on physicochemical, functional and thermal properties of potato and cassava starches. J. Food Eng..

[20] Hong J., Zeng X.A., Brennan C.S., Brennan M., Han Z. (2016). Recent Advances in Techniques for Starch Esters and the Applications: A Review. Foods.

[21] Chen Z., Schols H.A., Voragen A.G.J. (2004). Differently sized granules from acetylated potato and sweet potatostarches differ in the acetyl substitution patternof their amylose populations. Carbohydr. Polym..

[22] Das A.M., Singh G., Singh S., Riar C.R. (2010). Effect of acetylation and dual modification on physico-chemical, rheological and morphological characteristics of sweet potato (Ipomoea Batatas) starch. Carbohydr. Polym..

[23] Muhamedbegović B., Šubarić D., Babić J., Aćkar D., Jasić M., Keran H., Budimlić A., Matas I. (2012). Modification of potato starch. Technologica Acta.

[24] Punia S. (2020). Barley starch modifications: Physical, chemical and enzymatic-A review. Int. J. Biol. Macromol..

[25] BeMiller J.N., Welti-Chanes J., Serna-Saldívar S., Campanella O., Tejada-Ortigoza V. (2020). Resistant Starch. Science and Technology of Fibers in Food Systems.

[26] Alimi B.A., Workneh T.S., Oyeyinka S.A. (2017). Structural, rheological and in-vitro digestibility properties of composite corn-banana starch custard paste. LWT–Food Sci.Technol..

[27] Kiatponglarp W., Tongta S., Rolland-Sabaté A., Buléon A. (2015). Crystallization and chain reorganization of debranched rice starches in relation to resistant starch formation. Carbohydr. Polym..

[28] Trung P.H.B., Ngoc L.B.B., Hoa P.N., Tien N.N.T., Hung P.V. (2017). Impact of heat-moisture and annealing treatments on physicochemical properties and digestibility of starches from different colored sweet potato varieties. Int. J. Biol. Macromol..

[29] Xu M., Saleh A.S.M., Liu Y., Jing L., Zhao K., Wu H., Zhang G., Yang S.O., Li W. (2018). The changes in structural, physicochemical, and digestive properties of red adzuki bean starch after repeated and continuous annealing treatments. Starch/Stärke.

[30] Jayakody L., Hoover R. (2008). Effect of annealing on the molecular structure and physicochemical properties of starches from different botanical origins–A review. Carbohydr. Polym..

[31] Simsek S., Ovando-Martínez M., Whitney K., Bello-Pérez L.A. (2012). Effect of acetylation, oxidation and annealing on physicochemical properties of bean starch. Food Chem..

[32] Leszczyński W. (2004). Resistant starch–classification, structure, production. P. J. Food Nutr. Sci..

[33] Liu H., Guo X., Li W., Wang X., Iv M., Peng Q., Wang M. (2015). Changes in physicochemical properties and in vitro digestibility of common buckwheat starch by heat-moisture treatment and annealing. Carbohydr. Polym..

[34] Pinto V.Z., Vanier N.L., Deon V.G., Moomand K., El Halal S.L.M., Zavareze E.D.R., Lim L.T., Dias A.R.G. (2015). Effects of single and dual physical modifications on pinhão starch. Food Chem..

[35] Ashogbon A.O., Akintayo E.T. (2014). Recent trend in the physical and chemical modification of starches from different botanical sources: A review. Starch/Stärke.

[36] Sodhi N.S., Singh N. (2005). Characteristics of acetylated starches prepared using starches separated from different rice cultivars. J. Food Eng..

[37] Waduge R.N., Hoover R., Vasanthan T., Gao J., Li J. (2006). Effect of annealing on the structure and physicochemical properties of barley starches of varying amylose content. Food Res. Int..

[38] Golachowski A. (2003). Properties of acetylated starch obtained from SO2-treated starch milk. Electron. J. Pol. Agric. Univ..

[39] Gryszkin A., Zięba T., Kapelko M., Buczek A. (2014). Effect of thermal modifications of potato starch on its selected properties. Food Hydrocoll..

[40] Balcerowiak W. (2002). Różnicowa kalorymetria skaningowa. Materiały Trzeciej Szkoły Analizy Termicznej SAT.

[41] Zięba T., Kapelko M., Gryszkin A. (2007). Selected properties of potato starch subjected to multiple physical and chemical modifications. Polish J. Food Nutr. Sci..

[42] Kobryń J., Zięba T., Sowa S.K., Musiał W. (2020). Influence of Acetylated Annealed Starch on the Release of β-Escin from the Anionic and Non-Ionic Hydrophilic Gels. Pharmaceutics.

[43] Sanjivkumar B., Rajkumar D., Mallikarjun P., Karankumar B., Rao K.S. (2012). Development and method validation of Aesculus hippocastanum extract. Int. Res. J. Pharm..

